# Relative Efficacy of Checkpoint Inhibitors for Advanced NSCLC According to Programmed Death-Ligand-1 Expression: A Systematic Review and Network Meta-Analysis

**DOI:** 10.1038/s41598-018-30277-0

**Published:** 2018-08-06

**Authors:** Jinchul Kim, Jinhyun Cho, Moon Hee Lee, Joo Han Lim

**Affiliations:** Department of Hematology-Oncology, Inha University College of Medicine and Hospital, Incheon, Republic of Korea

## Abstract

Although currently available immune checkpoint inhibitors with similar but slightly different indications are recommended for patients with advanced non-small cell lung cancer (NSCLC), their effects by programmed death-ligand-1 (PD-L1) expression level are not yet known. This meta-analysis aims to assess the survival benefit and comparative efficacy of checkpoint inhibitors according to PD-L1 expression level: <1%, 1–49%, and ≥50%. We searched the MEDLINE, EMBASE, and Cochrane database through December 2017. A fixed-effect Bayesian network meta-analysis (NMA) was performed to estimate hazard ratios (HRs) for overall survival (OS) with 95% credible intervals (CrIs). Seven trials including 3688 patients were selected from among the 673 screened studies. Checkpoint inhibitor remarkably improved OS over chemotherapy in the PD-L1 ≥ 50% subgroup compared with the PD-L1 < 1% and PD-L1 1–49% subgroups. Atezolizumab, nivolumab, and nivolumab were the most effective agents for second- or later-line settings in the PD-L1 < 1%, PD-L1 1–49%, and PD-L1 ≥ 50% subgroups, respectively. PD-L1 expression ≥50% on tumor cells could be a reliable indicator that helps patient selection in view of cost-efficiency, and each checkpoint inhibitor reported to be the best agent by PD-L1 expression level could be carefully recommended in each PD-L1 expression subgroup.

## Introduction

Recent advancements in immune checkpoint inhibitors have revolutionised the treatment of incurable advanced non-small cell lung cancer (NSCLC) through targets such as the programmed death-ligand 1 (PD-L1) or its receptor, the programmed death-1 (PD-1) pathway. By blocking the immune escape mechanism of the tumor, PD-L1 or PD-1 inhibitors have reported fewer side effects and superior efficacy compared to those of conventional toxic chemotherapy^1–6^. Consequently, checkpoint inhibitors have been approved to replace chemotherapy as second-line treatment as well as the first-line treatment of patients with high PD-L1 expression on tumor cells^7^.

A useful biomarker for checkpoint inhibitors that could provide binary discrimination of responsiveness is urgently required and crucial, as only a small portion of the population with advanced NSCLC experiences long-term effects. PD-L1 expression on tumor cells is the most studied candidate to predict the efficacy of checkpoint inhibitor to date, although its clinical significance remains a topic of debate. Accordingly, all trials that compared checkpoint inhibitor with chemotherapy reported survival outcomes in the form of hazard ratio (HR) according to various PD-L1 cut-off levels^1–3,5,6,8–11^, and most studies reported an association between increased PD-L1 expression level on tumor cells and enhanced efficacy of PD-1 and PD-L1 inhibitors^1–3,5,6,9–13^.

Among the three available checkpoint inhibitors for advanced NSCLC patients, the PD-1 inhibitor pembrolizumab has been approved as a first-line therapy in patients with tumors harbouring PD-L1 expression ≥50% and as a second- or later-line treatment in patients with PD-L1 ≥1%^7^. The PD-1 inhibitor nivolumab and PD-L1 inhibitor atezolizumab have been approved as second- or later-line treatments regardless of PD-L1 expression^7^. In this situation, which has three recommended checkpoint inhibitors with a similar but slightly different clinical indication, a pooled analysis of survival data from currently available studies by PD-L1 expression level may provide insight into the role of PD-L1 expression on using checkpoint inhibitors and clinically useful evidence. Therefore, here we conducted a network meta-analysis (NMA) according to three PD-L1 expression level subgroups (<1%, 1–49%, and ≥50%) to evaluate the pooled effect of checkpoint inhibitors and assess the relative efficacy among the three checkpoint inhibitors in advanced NSCLC patients.

## Methods

### Systematic literature review

We carried out a systematic search of the literature from inception to December 28, 2017. Randomised controlled trials that compared a checkpoint inhibitor alone with chemotherapy in advanced NSCLC regardless of line of treatment were searched in MEDLINE, EMBASE, and the Cochrane Central Register of Controlled Trials. Searches were limited to human studies without language limitations. The following search phrases were used: (“immune checkpoint inhibitor” OR “PD-1” OR “PD-L1” OR “nivolumab” OR “pembrolizumab” OR “atezolizumab”) AND (“carcinoma, non-small-cell lung” OR “non-small cell lung cancer” OR “nsclc”). We also searched the meeting abstracts from the American Society of Clinical Oncology, European Society for Medical Oncology, and World Conference on Lung Cancer.

### Data extraction

We extracted the most extended follow-up data including updated survival analyses from the meeting abstracts in cases of multiple sources reported in the same trial. The following records were abstracted from each included study: trial name, year of publication, treatment details, line of treatment, PD-L1 diagnostic assay tool, clinical information on the study patients (age, never smoker, and histology) and the number of patients by three PD-L1 expression subgroups. The HRs with corresponding 95% confidence intervals (CIs) for overall survival (OS) were extracted from the included articles.

All included trials reported HRs and 95% CIs for OS in patients with expressions of PD-L1 < 1%, PD-L1 ≥ 50%, or PD-L1 ≥ 1%. To calculate HRs and 95% CIs for the PD-L1 1–49% subgroup of each trial, we assumed that combining log HR and its standard error for PD-L1 1–49% with log HR and its standard error for PD-L1 ≥ 50% by fixed-effect meta-analysis using the inverse-variance method could calculate HR and its 95% CI for PD-L1 ≥ 1%^14^. As we extracted HRs and 95% CIs for PD-L1 ≥ 1% and PD-L1 ≥ 50%, it was possible to calculate HRs and 95% CIs for PD-L1 1–49% of each trial. To test this hypothesis, we extracted and combined HRs that were reported in two subgroups with mutually exclusive property (e.g., male and female, non-squamous and squamous) in all included articles. The authors also checked whether calculated HRs corresponded to the reported HRs for the entire population, as PD-L1 1–49%, PD-L1 ≥ 50%, and PD-L1 ≥ 1% had the same property. From this approach, we identified that pooled HRs were nearly consistent with reported HRs for the overall population (with error ≤0.01). Two authors (J.K. and J.H.L.) abstracted the data independently using a predefined data sheet, and two other authors (J.C. and M.H.L.) resolved the discrepancies in the extracted data. Two reviewers (J.K. and J.C.) assessed the quality of the included studies using the Cochrane Collaboration risk-of-bias tool.

### Data synthesis and analysis

As included trials are well-designed randomised trials and similar in important ways, such as patient characteristics and outcome measurement, and due to the scarce number of trials consisting each edge of the network, a fixed-effect model was considered appropriate. A NMA using HRs for OS was conducted in the Bayesian framework using JAGS and the GeMTC package in R (https://drugis.org/software/r-packages/gemtc)^15,16^. To estimate relative HRs for OS, a Markov Chain Monte Carlo simulation was performed with 5,000 adaptations and 20,000 iterations of each of the four automatically generated Markov chains. After all simulations were performed, the NMA calculated the probability that each treatment would be best by calculating the percentage of simulations in which a certain treatment ranked first. Non-informative priors were chosen for the between-studies standard deviation and the relative effects of treatment. Heterogeneity in the network was evaluated via the standard deviation within each pairwise meta-analysis.

To provide more practical information in the clinical field and reduce the heterogeneity between studies that used the same checkpoint inhibitors, we also conducted a subgroup NMA including trials performed in second- or later-line settings.

## Results

**Figure 1:**
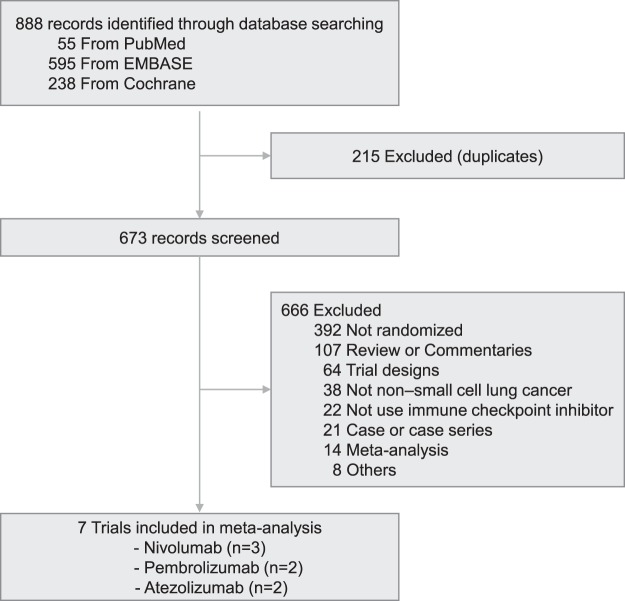
Trial selection flow diagram.

A total of 888 articles were identified in the initial database search. After the removal of 215 duplicate records, the titles and abstracts of 673 studies were screened. This meta-analysis included 3870 patients from seven randomised controlled trials (Fig. 1)^1–6,8–11,17^. Finally, 3688 patients were available for the analysis after excluding the patients for whom quantifiable PD-L1 expression information was not provided.

Table 1 shows the baseline characteristics of the seven included trials. Two trials^4,8,17^ were in first-line settings and five trials^1–3,5,6,9–11^ were in second- or later-line settings. Currently recommended chemotherapy regimens were used in all trials as the control group (platinum doublet chemotherapy for first-line therapy; docetaxel for second- or later-line therapy). Each checkpoint inhibitor had its own PD-L1 immunohistochemistry (IHC) diagnostic assay: Dako 28-8 for nivolumab^1,2,8,9^, Dako 22C3 for pembrolizumab^3,4,10,17^, and Ventana SP142 for atezolizumab^5,6,11^. Dako assays measured PD-L1 expression on tumor cells, whereas Ventana assays measured PD-L1 expression on both tumor cells and tumor-infiltrating immune cells. In the atezolizumab trials, the PD-L1 expression on ≥50% of tumor cells or ≥10% of immune cells was defined as “TC3 or IC3” and PD-L1 on <1% of tumor cells and <1% of immune cells as “TC0 and IC0”^5,6,11^. Accordingly, TC3 or IC3 was analysed as PD-L1 ≥ 50%, and TC0 and IC0 as PD-L1 < 1% in our study.

**Table 1:** Characteristics of the included studies comparing checkpoint inhibitor with chemotherapy.

					No. of Patients (%)					
							PD-L1 expression level			
Trial name	Line of Treatment	Treatment Comparison	PD-L1 diagnostic assay	Median Age (range)	Never smokers	Non-squamous	PD-L1 < 1%	PD-L1 1–49%	PD-L1 ≥ 50%	Follow-up Duration, mo
CheckMate 017^1^, 2015	Second or later	Nivolumab vs docetaxel	Dako 28–8 IHC assay	63(39–85)	17(6)	0(0)	106(39)	92(34)	27(10)	36.6 (minimum)
CheckMate 057^2^, 2015	Second or later	Nivolumab vs docetaxel	Dako 28–8 IHC assay	62(21–85)	118(21)	582(100)	209(36)	134(23)	112(19)	36.6 (minimum)
CheckMate 026^8^, 2017	First	Nivolumab vs platinum doublet chemotherapy	Dako 28–8 IHC assay	64(29–89)	59(11)	411(76)	0(0)	327(60)	214(40)	13.5 (median)
Keynote 010^3^, 2016	Second or later	Pembrolizumab vs docetaxel	Dako 22C3 IHC assay	63(56–69)	190(18)	724(70)	0(0)	591(57)	442(43)	19.2 (median)
Keynote024^4^, 2016	First	Pembrolizumab vs platinum doublet chemotherapy	Dako 22C3 IHC assay	65(33–90)	24(8)	249(81)	0(0)	0(0)	305(100)	25.2 (median)
POPLAR^5^, 2016	Second or later	Atezolizumab vs docetaxel	VENTANA SP142 IHC assay	62(36–84)	56(20)	190(66)	92(32)	148(52)	47(16)	20 (minimum)
OAK^6^, 2017	Second or later	Atezolizumab vs docetaxel	VENTANA SP142 IHC assay	64(33–85)	156(18)	628(74)	379(45)	326(39)	137(16)	21 (median)

IHC: immunohistochemistry; PD-L1: programmed death-ligand-1.

Publication bias could not be reported because of the small number of trials included in the pairwise comparisons. A good average of quality of included studies is provided in Supplementary Fig. S1. All trials reported a high risk of blinding of participants and personnel due to the open-label designs. Random sequence generation and allocation concealment were reported appropriately in the Keynote 010, POPLAR, and OAK trials. The CheckMate 017, CheckMate 057, and OAK trials reported an unclear risk of detection bias, which is evaluated by whether the outcomes of treatment are being assessed by a third independent reviewer. Attrition, reporting, and other biases were not detected in any of the trials.

### Efficacy of checkpoint inhibitors in overall analyses

**Figure 2:**
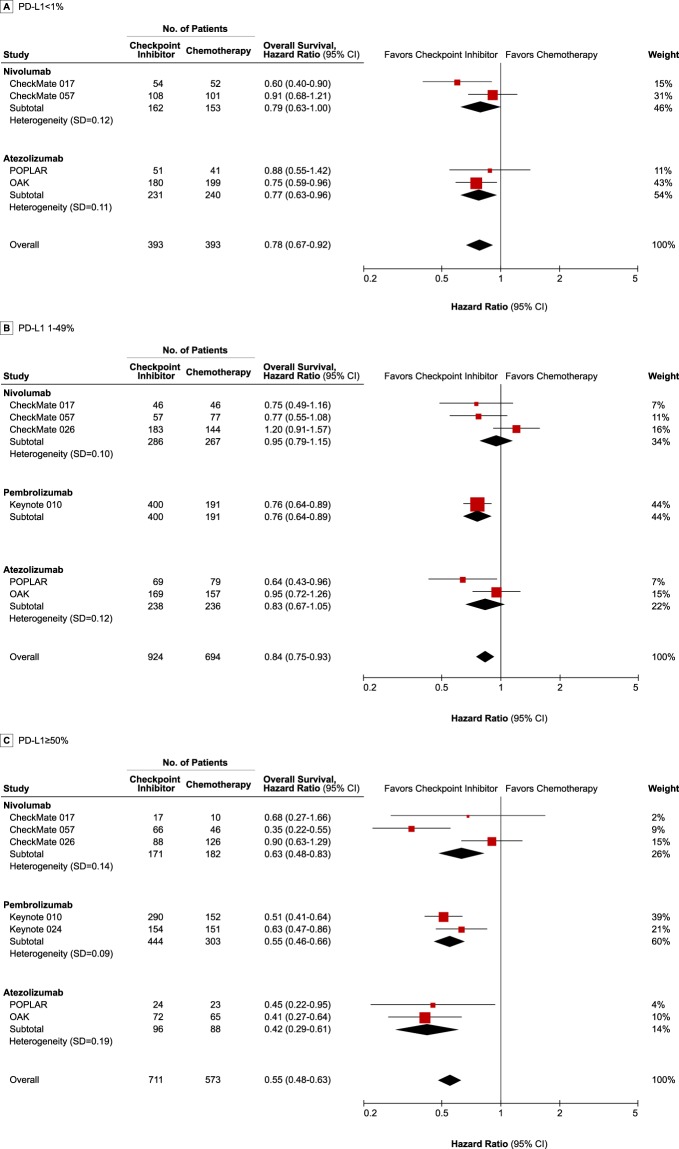
Forest plot of meta-analysis comparing checkpoint inhibitors vs chemotherapy for overall survival by PD-L1 expression. The size of the squares reflects the weight of the study in the meta-analysis. The effect size of individual trial represents the extracted hazard ratio and 95% confidence interval, and pooled effect-size represents the combined hazard ratio and 95% credible interval from meta-analysis. The combined effects were calculated with a Bayesian fixed-effect model. PD-L1: programmed death-ligand-1.

Checkpoint inhibitors improved OS compared with chemotherapy in all three subgroup patients with PD-L1 < 1% (HR, 0.78; 95% credible interval [CrI], 0.67–0.92), PD-L1 1–49% (HR, 0.84; 95% CrI, 0.75–0.93), and PD-L1 ≥ 50% (HR, 0.55; 95% CrI, 0.48–0.63) (Fig. 2). The Keynote 010 trial was not included in the PD-L1 < 1% group, while the Keynote 024 was included in only the PD-L1 ≥ 50% group because the Keynote 010^3,10^ and Keynote 024^4,17^ enrolled patients with the PD-L1 expression of at least 1% and 50%, respectively. For the same reason, the Checkmate 026 trial was not included in PD-L1 < 1%^8^. In patients with PD-L1 < 1%, both nivolumab (HR, 0.79; 95% CrI, 0.63–1.00) and atezolizumab (HR, 0.77; 95% CrI, 0.63–0.96) showed better survival outcomes than chemotherapy (Fig. 2A). Only pembrolizumab revealed statistically significant efficacy over chemotherapy (HR, 0.76; 95% CrI, 0.64–0.89) in patients with PD-L1 1–49% (Fig. 2B). All three checkpoint inhibitors had better efficacy than chemotherapy in patients with PD-L1 ≥ 50%, in whom atezolizumab showed the best efficacy (HR, 0.42; 95% CrI, 0.29–0.61). All relative effects of the checkpoint inhibitors and chemotherapy are shown in Supplementary Table S1.

### Relative efficacy of checkpoint inhibitors in second- or later-line settings

**Figure 3:**
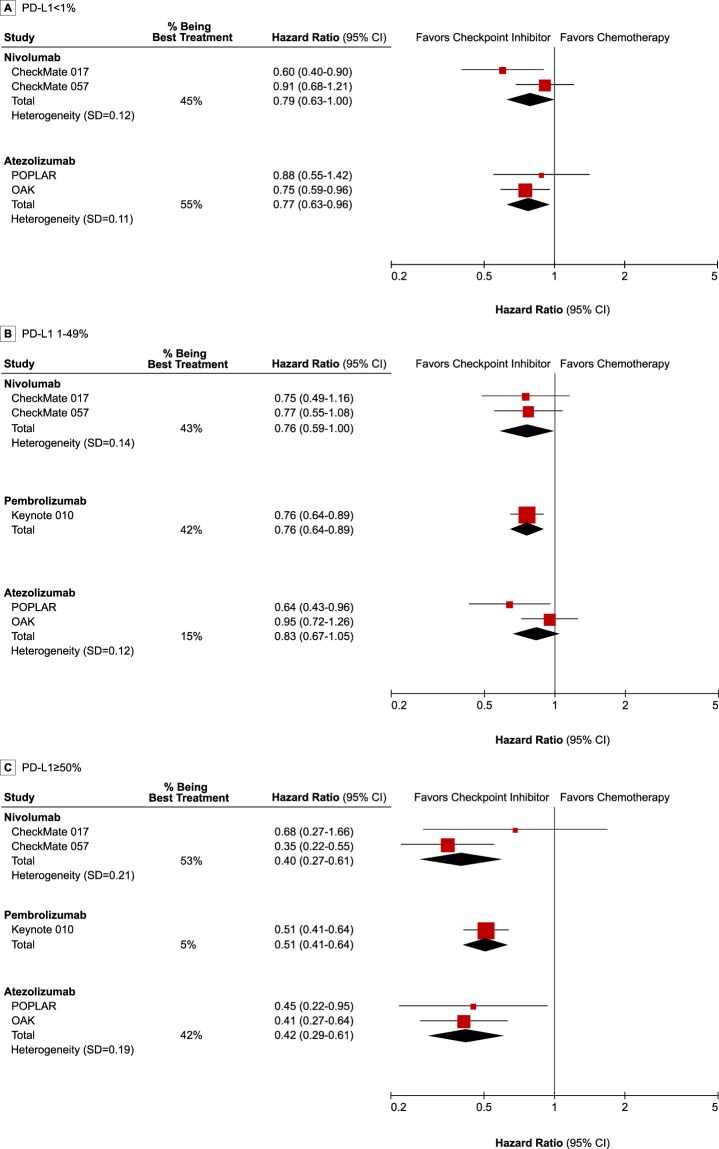
Forest plot of network meta-analysis results in second- or later-line settings by PD-L1 expression. The effect size of individual trial represents the extracted hazard ratio and 95% confidence interval, and pooled effect-size represents the combined hazard ratio and 95% credible interval from network meta-analysis. The combined effects were calculated with a Bayesian fixed-effect model. PD-L1: programmed death-ligand-1.

The comparative efficacy of checkpoint inhibitors and the probability of being the best treatment in second- or later-line treatment are presented in Fig. 3. The PD-L1 < 1% subgroup showed the same results as the overall analysis, because trials in the first-line settings enrolled patients with the PD-L1 expression on at least 1%^8^ or 50%^4,17^. In patients with PD-L1 < 1%, atezolizumab was the most effective treatment in 55% of the simulations versus nivolumab in 45%. In PD-L1 1–49%, nivolumab (HR, 0.76; 95% CrI, 0.59–1.00) and pembrolizumab (HR, 0.76; 95% CrI, 0.64–0.89) showed almost similar efficacy (43% and 42% of the probability of being best, respectively), while atezolizumab (HR, 0.83; 95% CrI, 0.67–1.05) did not demonstrate statistically significant better outcome compared to chemotherapy. All three agents showed impressive effects in PD-L1 ≥ 50%, and nivolumab (HR, 0.40; 95% CrI, 0.27–0.61, 53%), atezolizumab (HR, 0.42; 95% CrI, 0.29–0.61, 42%), and pembrolizumab (HR, 0.51; 95% CrI, 0.41–0.64, 5%) were ranked in order of the probability of being best in PD-L1 ≥ 50%. All relative effects of the checkpoint inhibitors and chemotherapy are shown in Supplementary Table S2 and the probabilities for each treatment to achieve each possible rank in Supplementary Table S3.

## Discussion

Several factors complicate decision-making for clinicians concerning the use of checkpoint inhibitors for treating advanced NSCLC. First, three different agents that have similar mechanisms of action are available for patients with advanced NSCLC. Second, each agent has a similar but slightly different indication of PD-L1 expression level. Third, although PD-L1 expression is approved as a companion or complementary diagnostic by the US Food and Drug Administration, the question of its clinical significance persists. In the situation mentioned above, we tried to evaluate the efficacy of checkpoint inhibitors by PD-L1 expression level and calculate the probability of each being the best treatment in second- or later-line settings through Bayesian simulations. This network meta-analysis demonstrated that the checkpoint inhibitor improved OS over chemotherapy in all three subgroups and a remarkably better effect was observed in the PD-L1 ≥ 50% subgroup than in the PD-L1 < 1% and PD-L1 1–49% subgroups, and provided information about the rank order of each treatment for second- or later-line settings.

The trend for a linear relationship between the PD-L1 expression level on tumor cells and the efficacy of checkpoint inhibitors have been reported in advanced NSCLC^1–3,5,6,9–13^. Based on this observation, the Keynote 024 trial comparing first-line platinum-based chemotherapy with pembrolizumab succeeded in reporting positive data by strictly selecting a predefined population with high PD-L1 expression on at least 50% of tumor cells^4,17^. However, the Checkmate 026, which compared first-line chemotherapy with nivolumab in patients with PD-L1 ≥ 1%, did not show a significant survival benefit of nivolumab even in those with PD-L1 ≥ 50%^8^. Various hypothetical factors may explain the difference in the results obtained from the Checkmate 026 compared with those from the Keynote 024 in the PD-L1 ≥ 50% subgroup. Among possible explanations, the most sound reasons may be a lack of power to detect an actual benefit of nivolumab in the Checkmate 026 due to a non-predefined design and an imbalance in the number of patients treated with nivolumab versus chemotherapy in PD-L1 ≥ 50% (88 vs 126)^18^. In our study, the PD-L1 ≥ 50% subgroup including 1284 patients showed a substantial benefit of using checkpoint inhibitors compared to the subgroup with PD-L1 < 1% or PD-L1 1–49%, suggesting that PD-L1 expression ≥50% would be a reliable indicator that helps with patient selection in view of cost-efficiency.

Current checkpoint inhibitors have their own IHC assay platforms, and different assay methods may lead to inappropriate result interpretation and treatment decisions. For this reason, efforts have been made to evaluate the comparability of various IHC assays^19–22^. Most studies reported that two PD-L1 IHC assays (Dako 22C3 and Dako 28–8) had similar performances for tumor cell staining of PD-L1, while SP143 showed less tumor cell staining than others^20–22^. Additionally, the SP143 assay quantified PD-L1 expression on tumor-infiltrating immune cells as well as on tumor cells. Therefore, the results for atezolizumab in this meta-analysis should be interpreted cautiously.

In our study, the distribution of patients by PD-L1 expression in trials^1,2,5,6^ that recruited patients regardless of PD-L1 expression was 786 (43%) in PD-L1 < 1%, 700 (39%) in PD-L1 1–49%, and 323 (18%) in PD-L1 ≥ 50% (Table). The findings were comparable to those of the study that reported on the prevalence of PD-L1 expression in patients investigated for enrollment in three pembrolizumab trials: the Keynote-001, -010, and -024^23^. In this study, 4784 patients were assessed for PD-L1 expression; 1596 (33%) had PD-L1 < 1% on tumor cells, 1832 (38%) had PD-L1 1–49%, and 1356 (28%) had PD-L1 ≥ 50%. From these statistics, it is possible to estimate the approximate distribution of patients with advanced NSCLC according to PD-L1 expression. The fact that advanced NSCLC has a relatively even distribution of PD-L1 expression in conjunction with the fact that there are similar clinical indications for checkpoint inhibitors could render it difficult for physicians to make clinical decisions. Our study might contribute to resolving this issue by dividing PD-L1 expression level into three subgroups with mutually exclusive categories and demonstrating the relative efficacies of checkpoint inhibitors and suggesting the best agents according to the subgroups.

The heterogeneity between first-line setting studies^4,8^ and those with the second- or later-line^1–3,5,6^ could occur from the factors that first-line chemotherapy could affect cancer immunogenicity^24^ and that trials performed in first-line settings allowed crossover from the chemotherapy arm to the checkpoint inhibitor arm at disease progression^4,8^, while second- or later-line setting trials did not allow crossover^1–3,5,6^. Indeed, preclinical data in a lung cancer mouse model demonstrated that the use of cyclophosphamide and oxaliplatin was associated with immune response stimulation, thereby producing a synergistic effect with checkpoint inhibitors^24^. In consistent with the preclinical data, our extracted data from the original studies showed that the second- or later-line checkpoint inhibitors had a superior impact to that of the first-line treatment compared with chemotherapy in the same PD-L1 expression subgroup. For this reason, we conducted a subgroup NMA including trials with second- or later-line settings to control the heterogeneity between studies with the same checkpoint inhibitors and investigate more useful data in the clinical field.

Although our study investigated the efficacy of checkpoint inhibitors as single agent, recently the study reporting first-line immune checkpoint inhibitor with cytotoxic chemotherapy for metastatic non-squamous NSCLC was published^25^. Pembrolizumab combination regimen, consisting pemetrexed, a platinum-based drug, and pembrolizumab, improved overall survival by all three subgroups of PD-L1 expression levels. The HRs of OS was 0.59 (95% CI, 0.38–0.92), 0.55 (95% CI, 0.34–0.90), and 0.42 (95% CI, 0.26–0.68) in PD-L1 expression of <1%, 1–49%, and >50%, respectively. All three subgroups showed better efficacy compared with single agent shown in our study, and PD-L1 > 50% group was also dominantly better than other PD-L1 expression groups. This result implicates that checkpoint inhibitor with chemotherapy could make a paradigm shift of first-line treatment for NSCLC.

Our study has several strengths. This-meta analysis was performed using the most updated survival analysis with a relatively sufficient follow-up duration of each trial, which supports the credibility of the data used in this analysis. Moreover, we separated PD-L1 expression levels into three subgroups with mutually exclusive categories, which could help with clinical decision-making processes using each patient’s PD-L1 status. Actually, previous study also analysed the relative effects of the checkpoint inhibitors in second- or later-line settings for advanced NSCLC^26^. However, this study evaluated the efficacies by PD-L1 expression level in an overlapping manner, such as ≥1%, ≥5%, ≥10%, and ≥50%. Our work differs from the study in that hazard ratios of overall survival in the range of 1–49% PD-L1 expression was computed in a robust way, providing more practical evidence. We also included 3688 patients with information about measurable PD-L1 expression level from seven randomised controlled trials, thereby securing adequate power to detect genuine differences. On the contrary, we faced several limitations during this study. First, the HRs and 95% CIs for the PD-L1 1–49% subgroup were calculated by the formula. Although we identified that this estimation could be a reasonable approximation, caution is needed when interpreting results of the PD-L1 1–49% group. However, for example, a survival analysis presenting the HR of the PD-L1 1–49% subgroup in the Keynote 010 trial^27^ that was shown at the 2016 ASCO annual meeting reported a pooled HR of 0.75 (95% CI, 0.62–0.91), and our study estimated the calculated HR of 0.76 (95% CI, 0.64–0.89) for the PD-L1 1–49% subgroup. It seems that there is little difference between two values considering the data from our study were retrieved from a longer follow-up period^10^, indicating our calculation could be a robust estimation. Second, as mentioned above, the VENTANA SP142 assay has slightly different properties compared to those of the other two tests, Dako 22C3 and 28-8. Third, other potential effect modifiers, such as previous radiotherapy history or imbalance in the number of patients between the checkpoint inhibitors and chemotherapy groups by PD-L1 expression subgroups were not considered. Despite these limitations, to the best of our knowledge, this NMA is the first study that performs a pooled analysis of seven checkpoint inhibitor trials with a focus on PD-L1 expression status.

In conclusion, for advanced NSCLC patients checkpoint inhibitors showed a more remarkable effect in the PD-L1 ≥ 50% subgroup than in the PD-L1 < 1% or PD-L1 1–49% subgroups. The subset NMA of the second- or later-line setting trials demonstrated the probabilities for each checkpoint inhibitor of being the best treatment by PD-L1 expression level. Based on our results, we carefully recommend atezolizumab, nivolumab, and nivolumab in patients with expressions of PD-L1 < 1%, PD-L1 1–49%, PD-L1 ≥ 50%, respectively.

## Electronic supplementary material

Supplementary Information
