# Supplementary material for: Relative Efficacy of Checkpoint Inhibitors for Advanced NSCLC According to Programmed Death-Ligand-1 Expression: A Systematic Review and Network Meta-Analysis
Source: Sci Rep. 2018 Aug 6;8:11738. doi: 10.1038/s41598-018-30277-0 (PMC6078964; doi:10.1038/s41598-018-30277-0)
Supplement: Supplementary file 1 — Supplementary Information [file 41598_2018_30277_MOESM1_ESM.pdf]

Supplementary Information

Relative efficacy of checkpoint inhibitors for advanced NSCLC according to programmed death-ligand-1 expression: a systematic review and network meta-analysis.

Jinchul Kim, Jinhyun Cho, Moon Hee Lee, Joo Han Lim.

Supplementary Figure S1. Risk of bias of included studies

Supplementary Table S1. Relative effects in pooled hazard ratios and 95% credible intervals by PD-L1 expression level

Supplementary Table S2. Relative effects in pooled hazard ratios and 95% credible intervals by PD-L1 expression level in second-or later-line settings

Supplementary Table S3. Rank probability in second- or later-line settings

Supplementary Figure S1. Risk of bias of included studies

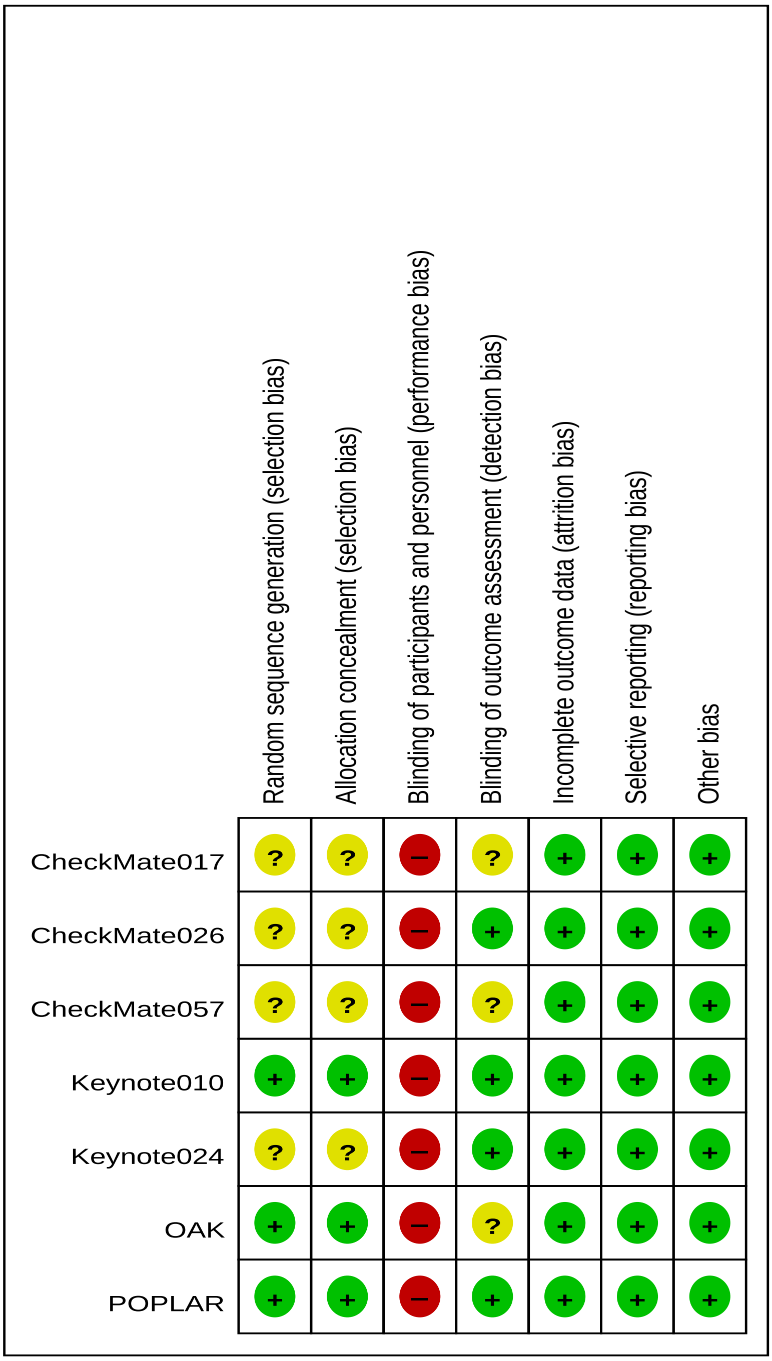

Supplementary Table S1. Relative effects in pooled hazard ratios and 95% credible intervals by PD-L1 expression level

A. PD-L1 < 1%

Chemotherapy	0.77 (0.63-0.96)	0.79 (0.63-1.00)
	Atezolizumab	1.02 (0.74-1.42)
		Nivolumab

B. PD-L1 1-49%

Chemotherapy	0.83 (0.67-1.05)	0.95 (0.79-1.15)	0.76 (0.64-0.90)
	Atezolizumab	1.14 (0.84-1.54)	0.91 (0.69-1.20)
		Nivolumab	0.80 (0.62-1.04)
			Pembrolizumab

C. PD-L1 ≥ 50%

Chemotherapy	0.42 (0.29-0.61)	0.63 (0.48-0.83)	0.55 (0.46-0.66)
	Atezolizumab	1.52 (0.95-2.40)	1.31 (0.87-1.97)
		Nivolumab	0.87 (0.63-1.20)
			Pembrolizumab

Each cell represents the effect of the column-defining intervention relative to the row-defining intervention. Effect estimates are presented as hazard ratios and 95% credible intervals are in parentheses.

Supplementary Table S2. Relative effects in pooled hazard ratios and 95% credible intervals by PD-L1 expression level in second-or later-line settings

A. PD-L1 < 1%

Chemotherapy	0.77 (0.63-0.96)	0.79 (0.63-1.00)
	Atezolizumab	1.02 (0.74-1.42)
		Nivolumab

B. PD-L1 1-49%

Chemotherapy	0.83 (0.67-1.05)	0.76 (0.59-1.00)	0.76 (0.64-0.90)
	Atezolizumab	0.92 (0.64-1.30)	0.91 (0.69-1.20)
		Nivolumab	1.00 (0.73-1.37)
			Pembrolizumab

C. PD-L1 ≥ 50%

Chemotherapy	0.42 (0.29-0.61)	0.40 (0.27-0.61)	0.51 (0.41-0.64)
	Atezolizumab	0.96 (0.55-1.66)	1.22 (0.79-1.87)
		Nivolumab	1.27 (0.80- 2.04)
			Pembrolizumab

Each cell represents the effect of the column-defining intervention relative to the row-defining intervention. Effect estimates are presented as hazard ratios and 95% credible intervals are in parentheses.

Supplementary Table S3. Rank probability in second- or later-line settings

A. PD-L1 < 1%

	Rank 1	Rank 2	Rank 3
Atezolizumab	0.55	0.44	0.01
Nivolumab	0.45	0.52	0.03
Chemotherapy	0	0.04	0.96

B. PD-L1 1-49%

	Rank 1	Rank 2	Rank 3	Rank 4
Atezolizumab	0.15	0.28	0.51	0.06
Nivolumab	0.43	0.31	0.24	0.02
Pembrolizumab	0.42	0.41	0.17	0
Chemotherapy	0	0	0.08	0.92

C. PD-L1 ≥ 50%

	Rank 1	Rank 2	Rank 3	Rank 4
Atezolizumab	0.42	0.42	0.16	0
Nivolumab	0.53	0.33	0.13	0
Pembrolizumab	0.05	0.25	0.71	0
Chemotherapy	0	0	0	1

Each cell represents the effect of the column-defining intervention relative to the row-defining intervention. Effect estimates are presented as hazard ratios and 95% credible intervals are in parentheses.
